# Surveillance Opportunities and the Need for Intersectoral Collaboration on Rabies in Sri Lanka

**DOI:** 10.1155/2019/7808517

**Published:** 2019-07-11

**Authors:** Pushpakumara Don Bamunusinghage Nihal, Ashoka Dangolla, Ranjani Hettiarachchi, Preeni Abeynayake, Craig Stephen

**Affiliations:** ^1^Department of Wildlife Conservation, Sri Lanka; ^2^Faculty of Veterinary Medicine and Animal Science, University of Peradeniya, Sri Lanka; ^3^Department of Animal Production and Health, Sri Lanka; ^4^Canadian Wildlife Health Cooperative, University of Saskatchewan, Saskatoon, Canada

## Abstract

Sri Lanka is progressing towards its goal of eliminating human rabies. This goal rests on programs designed to limit canine rabies, which in turn requires a combination of targeted dog rabies control and a better understanding of the movement of the virus between domestic animals, people, and wildlife. Coordinated and integrated surveillance of the disease between human and animal health sectors underpins successful rabies elimination. Our objective was to review surveillance data from 2005 to 2014 to assemble the first multispecies synthesis of rabies information in Sri Lanka and, in doing so, assess needs and opportunities for a One Health approach to rabies surveillance in the country. Our descriptive epidemiological findings were consistent with other studies showing a decline in human cases, endemic and unchanging numbers of dog cases, a relationship between human density and the occurrence of human and animal cases, and significant gaps in understanding trends in rabies incidences in livestock and wildlife. Assessing the trends in the data from the three government organizations responsible for rabies surveillance was difficult due to lack of information on animal population sizes, unquantified sampling biases due to inequities in access to diagnostic capacities, regulatory and administrative barriers, and a continued reliance on clinical means to establish a diagnosis. The information required for a comprehensive rabies control programme was not standardized or consistent, was not in one place, showed significant gaps in completeness, and was not amenable to routine and rapid analysis. Achieving rabies elimination in Sri Lanka would benefit from harmonization of diagnostic and information management standards across animal and human health sectors as well as equitable access to diagnostic capacity for all regions and species.

## 1. Introduction

Rabies is endemic in Sri Lanka but eliminating human rabies is within grasp. The number of human cases has been steadily decreasing from 370 deaths per year in 1970 to 19 in 2014 [1] (more recent statistics were unavailable at the time of writing this paper). Human deaths are primarily from rabid dog bites which account for 93-96% of all animal bites in people in the country [2]. The disease creates an economic burden due to heavy costs of human postexposure treatment, diagnosis, surveillance, and animal immunization [3]. Rabies elimination and advocating and supporting intersectoral approaches for communicable disease preparedness and response were both strategic areas identified in the World Health Organization 2012–2017 Sri Lanka Country Cooperation Strategy [4]. Strengthening surveillance is a priority to attaining zero human deaths from rabies in Sri Lanka [5].

Sri Lanka is an island nation located in the Indian Ocean to the southwest of the Bay of Bengal. Sri Lanka has the advantages of no physical connections to neighboring countries which facilitates rabies biosecurity and elimination. Rabies elimination requires an interdisciplinary approach because rabies control does not fit into the domain of any single administrative agency. Sri Lankan policy allocates dog rabies control to the Ministry of Health (MOH) and Ministry of Local Government. The animal health sector has historically had only minimal involvement in rabies control activities. There is no laboratory results based nationwide animal rabies surveillance system. The Department of Animal Production and Health (DAPH) conducts animal rabies surveillance based on clinical signs and reports their findings to the World Organization for Animal Health (OIE). The impact of rabies on livestock production is unknown and the epidemiology of the disease in wildlife is not studied and uncertain. Ministries dealing with animal health are focused mainly on economically important agricultural animal diseases affecting livestock productivity. Public concern about rabies control has increased conversations about the social responsibility and role of the Ministry of Animal Production and Health and MOH in rabies control. Poor surveillance of the disease in animals and weak coordination between the human and animal health sectors can impede rabies elimination [6].

Rabies circulates in two epidemiological cycles in Sri Lanka: an urban cycle involving maintenance of infection in dog populations and a sylvatic cycle involving wild animals [2]. There is a possibility of spill-over of rabies virus from dogs to wildlife and vice versa. Mongoose (*Herpestes vitticollis, Herpestes brachyurus, Herpestes edwardsii, and Herpestes smithii*), jackals (*Canis aureus),* Giant squirrel (*Ratufa macroura)*), and Palm civet cat (*Paradoxurus zeylonensis, Paradoxurus hermaphrodites, and Viverricula indica*) have been identified as wildlife reservoirs of rabies in Sri Lanka [7]. Domestic cats have also been found to be transmitters of rabies and domestic animals such as cattle, buffalo, pigs, sheep, and goats have been rabies positive [8]. The most important rabies transmitter species is the dog while the other animals play minor roles.

Our objective was to review existing data from 2005 to 2014 to attempt to assemble the first multispecies description of rabies surveillance information in Sri Lanka and, in doing so, assess needs and opportunities for a One Health approach to rabies surveillance in the country.

## 2. Methods

Data from all regions of Sri Lanka between 2005 and 2014 was obtained from the Public Health Veterinary Service (PHVS), Medical Research Institute (MRI), and the Department of Animal Production and Health (DAPH), the 3 government agencies with authority and responsibility for rabies surveillance and diagnosis in people and animals. MRI is the central laboratory for rabies diagnosis and two other peripheral laboratories in University of Peradeniya and Teaching Hospital Karapitiya help to diagnose rabies in animals. This time period was selected as stray dog control methods shifted from killing to surgical and chemical sterilization after the TSUNSMI disaster in 2004 and complete data sets were only available up to 2014 at the time of this investigation.

The PHVS and MRI had data on fluorescent antibody test (FAT) confirmed human cases as well as human cases strongly suspected as rabies based on clinical signs (fan signs positive, paralysis, other nervous signs and having the history of animal bite). Suspected rabies deaths were submitted for FAT testing and any negative findings resulted in elimination of clinical cases from the rabies registry. Data collected from the PHVS and MRI were cross-referenced with the data available at epidemiology unit of the MOH. Demographic and descriptive data were sought for each case including district and the year of diagnosis.

Animal rabies data was generated by the PHVS, MRI, and DAPH, depending on the species and situation. Laboratory confirmation required the detection of Negri body test and/or positive FAT results. Those which lacked Negri bodies despite the rabies suggestive history were subjected to FAT before issuing the report. Rabies suspected cases were based on clinical signs determined by field veterinary officers. Cases were categorized by species, district in which the animal was found, and year in which the cases were reported. MRI and PHVS recorded all the samples which were sent for laboratory diagnosis and their test results. DAPH recorded cases clinically diagnosed by the field veterinary surgeons. PHVS recorded all the laboratory confirmed cases from MRI, University of Peradeniya, and Karapitiya Laboratories.

Summary statistics were calculated for numbers and proportions of rabies positive cases, by categories of other variables. Cumulative incidence (CI) estimates per 100,000 populations were made for each district for dog and human rabies cases. Human population data were collected from Department of Census and Statistics website (https://www.census.gov/ and http://www.statistics.gov.lk/) for each of the 25 districts in the country. Dog population for each of the 25 districts were estimated rather than measured using methods that assumed a dog to human ratio of 8:1 [9, 10] as no canine population census was available from all districts for all time periods of the study. Number of rabies cases of both human and dog in each district during the 10 years period was graphically presented on choropleth maps using Arc GIS (Imgrd 10.40.63.198)

Insights into the organization and operation of rabies programs were obtained from review of legislated responsibilities of the government agencies responsible for rabies control and surveillance and review of the records by the primary author (NP) as well as through conversations with individuals responsible for delivering these programs.

## 3. Results

### 3.1. Descriptive Epidemiology

Between 2005 and 2014, a total of 9,179 human and animal rabies cases were reported, of which 7,417 (80.8%) were laboratory confirmed and 1762 (19.2%) were clinically diagnosed by medical officers or field veterinarians. Out of the total number, dogs provided 6788 cases (74.0%), livestock 1197 (13.0%), cats 663 (7.2%), wild animals 63 (0.7%), and people 467 (5.1%).  Table 1 summarizes the laboratory confirmed and clinically diagnosed rabies cases and distribution of their percentages in each species during the study period.

A total of 15,251 samples were submitted for laboratory diagnosis of which 7417 (48.6%) were confirmed based on the presence of Negri bodies, a positive FAT or both. Brain samples were submitted for laboratory testing for clinically suspected animal or human cases and from animals that had bitten people. Most submissions came from dog (n = 10,513, 68.9%) followed by cats (n = 3273, 21.5%) and human brains (n = 374, 2.4%). The positivity rate was highest among human samples (305/374, 81.5%). The second highest positivity rate was from cattle (120/167, 71.8%). The highest number of sample submission was from dogs; their positivity rates were 59.2%. All the bat samples were negative (9/15,251, 0.06%).  Table 2 summarizes the frequency of total tested, positive, negative and decomposed samples from human, companion animals, livestock and wildlife submissions to the MRI and PHVS.

A total of 8712 animal rabies cases were reported of which 1600 (15.4%) were clinically diagnosed. Out of the clinically diagnosed cases, the highest number was from cattle (n = 795). The second highest was from dogs (n = 561). There were 63 laboratory confirmed wild animal rabies cases but with no clinically diagnosed wildlife rabies reported. The clinically diagnosed animal rabies cases were only from domestic animal cases reported by DAPH. Of the 20 species of animal contributing samples for diagnosis, dogs had the highest percentages of positives (87.55%, n = 6227).

**Figure 1:**
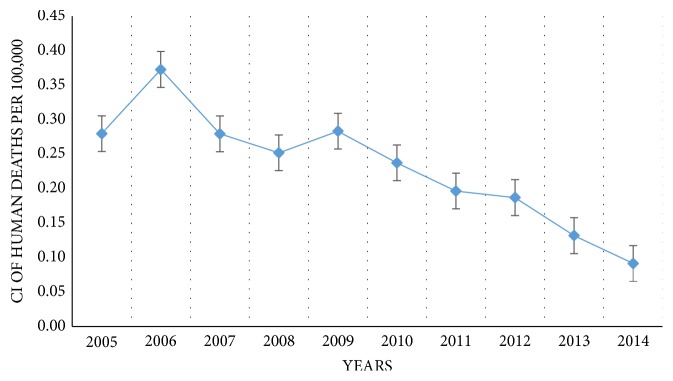
Annual cumulative incidences of human deaths from rabies in Sri Lanka per 100,000 population between 2005 and 2014.

**Figure 2:**
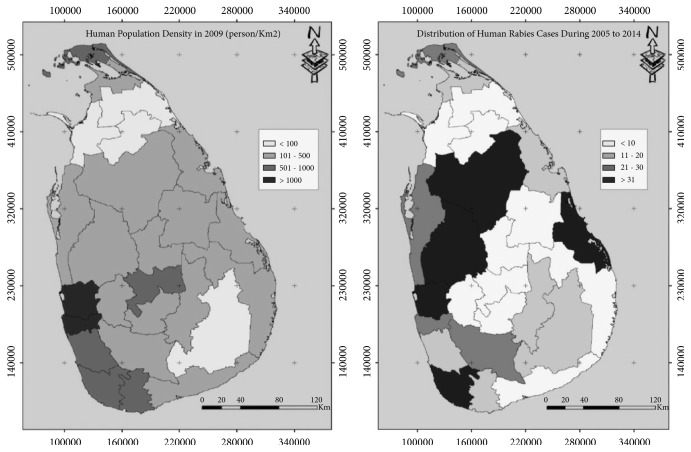
Geographic differences in human population density (persons/km2) (left) and number of human rabies cases recorded in national surveillance databases (right) between 2005 and 2014 in each district of Sri Lanka.

The 467 reported human rabies cases included both laboratory confirmed (65.3%) and clinically diagnosed cases (34.7%). Numbers of human deaths and cumulative incidence declined throughout the study period (Figure 1). There were marked differences in human population number and rabies deaths in each district. The highest number of deaths (n = 55) were reported from the Gampaha, second highest (n = 45) from the Kurunegala, and the lowest (n = 4) from Nuwara Eliya district.  Figure 2 visualizes the human population density in each district and the total human deaths due to rabies during the 10 years period. High human population densities were observed in Colombo, Gampaha, Kalutara, Galle, Matara, Kandy, and Jaffna districts. High numbers of human deaths due to rabies were reported in Gampaha, Kurunegala, Anuradhapura, Galle, and Batticaloa districts. The highest human population density was from Colombo district throughout the study period which was 3553.25 people per square kilometer in 2005. The lowest human population density was in Mullaitivu which was 66.66 per square kilometer in 2005

There was no noticeable seasonal pattern to the occurrence of monthly cases. However, the highest numbers of reported animal rabies cases were generally seen in January to March (average n = 580) and August to October (average n = 563). Laboratories received similar numbers of submissions each month of the year. The number and rate of human rabies decreased over the study period despite an average of 622.7 dog cases per year that showed no signs of a consistent decrease.

**Figure 3:**
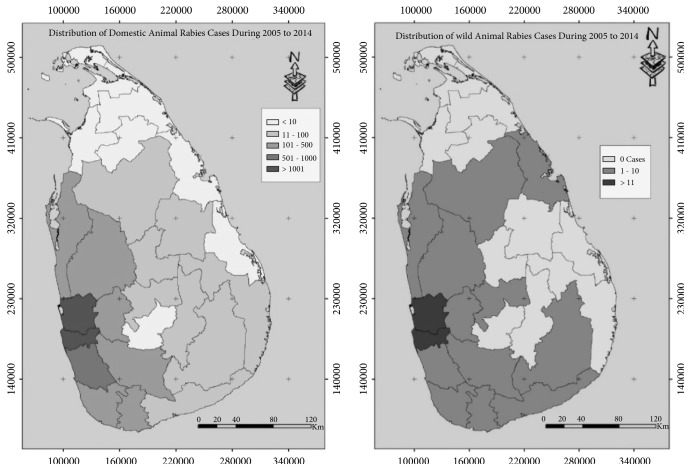
Geographic differences in the number of domestic animal rabies cases (left) and wild animal cases (right) recorded in national surveillance databases between 2005 and 2014 in each district of Sri Lanka.

Animal rabies cases were distributed throughout the country. The highest number of cases were reported from Colombo (n = 1920) followed by Gampaha (n = 1747) districts in the western province of Sri Lanka. No cases were reported from Mullaitivu district (n = 0).  Figure 3 presents the distribution of domestic and wild animal cases of rabies.

### 3.2. Surveillance System Attributes

Human and animal rabies data were distributed between the MRI, PHVS, DAPH, and MOH. Animal rabies laboratory data were collected by MRI and sent monthly to the PHVS. Field Veterinary Surgeons in the DAPH report clinically diagnosed rabies cases through their monthly report to the DAPH. Subsequent reports on the monthly distribution of animal rabies cases were submitted to the OIE via the World Animal Health Information System (WAHIS). DAPH quarterly epidemiology bulletins included information on clinically diagnosed animal rabies cases. However this information was not integrated with human rabies data. Human rabies cases, both laboratory confirmed and clinically diagnosed, were reported to the epidemiology unit of the MOH which then further investigated the patient history and animal bite history. Public Health Inspectors in charge of rabies control in relevant district reported cases to PHVS but did not cross check with the epidemiology unit not including animal rabies cases. Laboratory confirmed rabies cases were sent to PHVS and epidemiology unit of the MOH directly by MRI.

Wildlife surveillance activities were strongly limited by regulatory restrictions and absence of communication between public health, domestic animal health, and wild animal health sectors. Even though regional wildlife veterinarians and wildlife field officers routinely handled diseased and dead wildlife, systematic investigation, recording, and reporting of such incidences were lacking. This was attributed to a lack of infrastructure to collect and submit samples and unavailability of laboratory facilities. Absence of trained and skilled staff, work overload, and less attention to preventive health within Department of Wildlife Conservation were identified as constraints.

The quality of data on rabies control was not uniform across all institutes. Several pieces of information were missing regarding individual cases. Specially, information such as age and sex of the animals and previous vaccination history were missing in most of the rabies confirmed cases. The geographical location of confirmed rabies cases was only recorded at the district level. Therefore, most cases could not be attributed to the specific geographical location of origin or reporting unless specifically informed by the MRI. In hospitals, where postexposure treatment is performed, information on animal bites and other relevant data were recorded but such information was very rarely integrated with case surveillance data for program planning or evaluation purposes.

The format in which surveillance and rabies control data were recorded in administrative offices/institutes was not similar. Several opportunities of human errors and bias in data recording were noted, including the use of paper records in some cases. The recorded data were not regularly monitored, cross checked, or validated.

Data on dog rabies control efforts (vaccination, sterilization and stray dog capture and removal) were collected at regional level and sent to provincial level, then to the PHVS at the national level. The PHVS was responsible for storage and analysis of this data and produced reports quarterly. Information and data regarding dog rabies control efforts were recorded on paper and had never been cross checked for their validity. Some NGOs and private veterinary practitioners conducted dog vaccinations and surgical and medical sterilization campaigns. These activities are not reported because it was not a regulatory requirement and there was no central capacity to collect those data.

## 4. Discussion

Several factors reduced confidence in descriptive epidemiology of human and animal rabies cases based on surveillance data retained by government agencies. Firstly, dog population numbers were not determined by census and the population size of many wild species is unknown. This precludes the calculation of rates of disease which in turn complicates efforts to dissect the effects on observed trends due to rabies control programs versus changes in animal population abundance and distribution. A decline in the proportion of test positive dog samples was observed by others [2, 8, 11] but our results showed an increasing positivity rate, from 50.5% positivity rate in 2005 to 63% positivity rate in 2014. This increase positivity rate may be due to experience of laboratory technicians, adoption of high quality techniques, and training on detection of Negri body on brain smears and/or changes in the nature of samples being submitted for testing. Dog mass vaccination programmes during the past few decades and changing dog population size due to fertility and stray animal control would be expected to reduce the positivity rate of dog rabies. But these indicated benefits could have been offset by limitations of the dog vaccination programme to maintain herd immunity in the dog population. The human population data was based on census results, allowing more confident calculation of cumulative incidence.

The north and east part of the country had low numbers of reported domestic animal, wild animals, and human rabies cases except in the Batticaloa district. This part of the country was affected by decades of civil war which influenced both human population size and access to veterinary and medical services. Both this and other studies [2, 7] found more rabies cases in the western province which also has more health care workers and facilities compared to other provinces in the country. This trend could also be due a lower density of stray dogs in the north and east given that Matsumoto et al. [2] concluded that rabies in Sri Lanka directly correlates with dog population density.

There were several opportunities for detection of biases in the surveillance data. Access to diagnostic services was not equally distributed all over Sri Lanka due to skewed distribution of diagnostic laboratories and diagnosticians and logistic challenges in obtaining and submitting samples. Case numbers were higher near regions with higher human population density. Colombo, Gampaha, and Kalutara districts of the western province had higher human densities and therefore more opportunities for people to detect and report sick animals. The main investigating laboratory is situated in the Colombo district in the western province which makes it easier for samples to be quickly and cheaply shipped within the western province. Sample transportation for submission to the centrally located MRI from remote areas is costly and time consuming. The unpleasant nature of handling dead animals and lack of trust of the laboratory results could have affected willingness to obtain and submit samples. Sawford et al. [12] found that participation in surveillance efforts by DAPH field veterinarians varied between districts, further affecting the probability of sample submission.

Most of the time only urban wildlife was subjected to rabies diagnosis. Submission of samples from wildlife habitat rich districts was lacking due to various reasons including lack of interest among public. It is also possible that scavenging among wild animals, difficulty of capture and legal issues regarding wild animal transportation may also have played roles. Mongoose, giant squirrels, and palm cats were the most frequent rabies positive wild animals. These animals are likely to inhabit urban and periurban areas and have higher opportunity to be in contact with dogs. There is a very small cohort of veterinarians with training or authority to manage and assess wildlife diseases in Sri Lanka. This may have implications for rabies elimination. For example, despite the lack of rabies positive bats in this study, bats were found to carry a new strain of Lyssa virus in Sri Lanka, without evidence of interspecies transmission [13]. The rabies situation in wild animals cannot be described using the current surveillance programme. This will become a more pressing issue as Sri Lanka gains control over human and domestic dog rabies and needs to seek other animal reservoirs to target for rabies elimination.

The total number of samples from livestock tested for rabies was much less compared to dogs and cats. This may be due to difficulties in sample submission for laboratory diagnosis due to large size of the samples (animal heads) and difficulties in packing. Out of the total tested, the highest proportions of positive sample submitted were from cows (71.9%). Unavailability of peripheral laboratories for confirmation of clinically suspected rabies among livestock is a substantial problem. According to Wasi et al. [11], the total numbers of sample submissions decrease when exposed people are more willing and able to get postexposure treatment and, therefore, reducing the priority of animal case confirmation.

There was a possibility of misclassification bias in a significant proportion of the cases since a relatively high proportion of human and animal cases were based on clinical diagnosis. Most animal cases depended on the Negri body detection test which is known to be neither sensitive nor specific [14]. FAT was done only on Negri body negative samples. Low numbers of positive wild animal samples may be due to poor knowledge of clinical signs of rabies in wild animals.

Despite these limitations, the trends seen in this study are consistent with past characterizations of rabies in Sri Lanka [2, 7, 8]. Sri Lanka is not dissimilar to many countries where authority for animal aspects of a zoonotic disease control and human aspects are subdivided by regulatory authority between different agencies. Sri Lanka also faces the challenges of many lower- and middle-income countries in attaining the necessary resources to fully implement integrated surveillance systems. Animal rabies control competes with other priorities in animal health departments emphasizing the need for accurate data to determine the cost and impact of the disease. Our results suggest that achieving rabies elimination in Sri Lanka would benefit from harmonization of diagnostic and information management standards across animal and human health sectors as well as equitable access to diagnostic capacity for all regions and species. Investigators using rabies data in a collaborative manner should take into account the varying pressures and biases that limit the capacity to associate disease trends with control efforts.

## Figures and Tables

**Table 1:** Reported rabies cases in different species in Sri Lanka from 2005 to 2014 based on data provided by the Medical Research Institute and the Department of Animal Production and Health and PHVS in Sri Lanka.

Species		Laboratory confirmed (%)	Clinically diagnosed (%)	Total (%)
Common name	Scientific name			
Dog	*Canis familiaris*	6227 (67.83)	561 (6.11)	6788 (73.95)
Cattle	*Bos Taurus*	120 (1.30)	795 (8.66)	915 (9.96)
Cat	*Felis catus*	661 (7.20)	2 (0.02)	663 (7.22)
Human	*Homo sapiens*	305 (3.32)	162 (1.76)	467 (5.08)
Goat	*Caprine spp.*	25 (0.27)	208 (2.26)	233 (2.53)
Buffalo	*Bubalus spp.*	8 (0.09)	15 (0.16)	23 (0.25)
Mongoose	*Herpestes brachyurus*	20 (0.21)	0 (0.00)	20 (0.21)
Pig	*Sus scrofa domesticus*	7 (0.08)	6 (0.06)	13 (0.14)
Sheep	*Ovis aries*	0 (0.00)	13 (0.14)	13 (0.14)
Giant Squirrel	*Ratufa macroura*	10 (0.10)	0 (0.00)	10 (0.10)
G/Mongoose	*Herpestes edwardsii*	8 (0.09)	0 (0.00)	8 (0.09)
Palm cat	*Paradoxurus hermaphroditus*	8 (0.09)	0 (0.00)	8 (0.09)
Squirrel	*Funambulus palmarum*	7 (0.08)	0 (0.00)	7 (0.08)
Monkey	*Semnopithecus spp & Macca spp.*	3 (0.03)	0 (0.00)	3 (0.03)
Bandicoot	*Bandicota bengalensis*	2 (0.02)	0 (0.00)	2 (0.02)
Civet cat	*Viverricula indica*	2 (0.02)	0 (0.00)	2 (0.02)
Wild cat	*Felis chaus*	2 (0.02)	0 (0.00)	2 (0.02)
Guinea pig	*Cavia porcellus*	1 (0.01)	0 (0.00)	1 (0.01)
R/Mongoose	*Herpestes smithii*	1 (0.01)	0 (0.00)	1 (0.01)

**Table 2:** Number and disposition of human and animal samples received for laboratory diagnosis for rabies between 2005 and 2014 in Sri Lanka, based on data provided from the Medical Research Institute and the Public Health Veterinary Service of Sri Lanka.

Category of sample	Animal species	Total sample received	Positive (%)	Negative (%)	Decomposed
Human	Human	374	*305 (81.5)*	52 (13.9)	17

Companion animal	Dog	10513	6227 (59.2)	3622 (34.4)	664

	Cat	3273	661 (20.2)	2434 (74.4)	178

*Sub total*			*6888 (49.9)*		

Domestic animal	Cattle	167	120 (71.8)	45 (26.9)	2

	Rabbit	60	0 (00.0)	58 (96.7)	2

	Goat	42	25 (59.5)	16 (38.1)	1

	Pig	14	7 (50.0)	4 (28.6)	3

	Buffalo	12	8 (66.7)	4 (33.3)	0

*Sub total*			*160 (54.2)*		

Wild animal	Squirrel	308	7 (2.3)	293 (95.1)	8

	Giant Squirrel	135	10 (7.4)	115 (85.2)	10

	Mongoose	123	29 (23.5)	91 (73.9)	3

	Rat	97	0 (00.0)	92 (94.8)	5

	Toque monkey and Gray langurs	48	3 (6.2)	42 (87.5)	3

	Palm cat	41	8 (19.5)	30 (73.2)	3

	Civet cat	14	2 (14.3)	12 (85.7)	0

	Bandicoot	11	2 (18.2)	9 (81.8)	0

	Bat	9	0 (00.0)	9 (100)	0

	Wild cat	5	2 (40.0)	3 (60.0)	0

*Sub total*		791	*63 (7.9)*		

Laboratory animal	Guinea pig	5	*1 (20.0)*	3 (60.0)	1

## Data Availability

Data were extracted from information available through relevant government departments in Sri Lanka as described in the text.
